# Cerium/Ascorbic Acid/Iodine Active Species for Redox Flow Energy Storage Battery

**DOI:** 10.3390/molecules26113443

**Published:** 2021-06-05

**Authors:** Tzu-Chin Chang, Yu-Hsuan Liu, Mei-Ling Chen, Chen-Chen Tseng, Yung-Sheng Lin, Shu-Ling Huang

**Affiliations:** 1 Ph.D. Program in Materials and Chemical Engineering, National United University, Miaoli 36063, Taiwan; m0514007@gm.nuu.edu.tw (T.-C.C.); linys@nuu.edu.tw (Y.-S.L.); 2 Department of Chemical Engineering, National United University, Miaoli 36063, Taiwan; gre2625@gmail.com (Y.-H.L.); u0314006@gmail.com (C.-C.T.); 3 Department of Electrical Engineering, National United University, Miaoli 36063, Taiwan

**Keywords:** Ce/ascorbic acid/I RFB, electroless plating, sol–gel, C–TiO_2_–PdO composite electrode, electrocatalytic, Nafion 117–SiO_2_–SO_3_H membrane

## Abstract

In this study, we developed a novel cerium/ascorbic acid/iodine active species to design a redox flow battery (RFB), in which the cerium nitrate hexahydrate [Ce(NO_3_)_3_·6H_2_O] was used as a positive Ce^3+^/Ce^4+^ ion pair, and the potassium iodate (KIO_3_) containing ascorbic acid was used as a negative I_2_/I^−^ ion pair. In order to improve the electrochemical activity and to avoid cross-contamination of the redox pair ions, the electroless plating and sol–gel method were applied to modify the carbon paper electrode and the Nafion 117 membrane. The electrocatalytic and electrochemical properties of the composite electrode using methanesulfonic acid as a supporting electrolyte were assessed using the cyclic voltammetry (CV) test. The results showed that the Ce (III)/Ce (IV) active species presented a symmetric oxidation/reduction current ratio (1.09) on the C–TiO_2_–PdO composite electrode. Adding a constant amount of ascorbic acid to the iodine solution led to a good reversible oxidation/reduction reaction. Therefore, a novel Ce/ascorbic acid/I RFB was developed with C–TiO_2_–PdO composite electrodes and modified Nafion 117–SiO_2_–SO_3_H membrane using the staggered-type flow channel, of which the energy efficiency (EE%) can reach about 72%. The Ce/ascorbic acid/I active species can greatly reduce the electrolyte cost compared to the all-vanadium redox flow battery system, and it therefore has greater development potential.

## 1. Introduction

Electrochemical energy storage technology has been developed for many energy storage methods over recent years. Among them, the development of the redox flow battery (RFB) has received the most attention. The RFB has been developed for large-scale energy storage systems (KWh–MWh) during the last decade to address the power instability of renewable energy resources (e.g., wind power and solar power) because of climate change. RFBs have been regarded as suitable for large-scale energy storage because of their modular design, good scalability, flexible operation, high storage capacity and efficiency, long operating life, safety, and environmentally friendly properties [1, 2, 3, 4, 5]. E. Sánchez-Díez et al. provided a review of the status of and perspective towards sustainable stationary energy storage of the RFB, which can potentially fulfill cost requirements and enable large-scale storage [1]. A. Clemente et al. reviewed the concept of the RFB, such as the composition and operation, including battery sizing, main existing applications and installations, mathematical models, and control and supervision [2]. L. Sanz et al. proposed the novel electrochemistry of the all-copper system, which is well suited to application in RFB technologies [3]. F. Pan et al. reviewed different categories of storage active species for RFB applications, such as inorganic ions (metal complexes) and metal-free organic compounds (e.g., polysulfide/sulfur and lithium) storage and suggested the future development of redox species towards higher energy density [4]. S. N. Garcia et al. synthesized four different benzyl-morpholino hydroquinone derivatives as potential redox active species [5]. The all-vanadium redox flow battery (all-VRFB) is one of the most promising technologies for mid- to large-scale application (KW–MW) and has the advantages of fast response time, flexible design, high cycle life, and non-flammable and non-explosive properties. M. Skyllas-Kazacos et al. first put forward the VRFB in the 1980s [6]. M. Pugach et al. proposed a new methodology for estimation of the key characteristics for a commercial scale (5 KW/15 KWh) VRFB at different operating conditions [7]. T. Sarkar et al. approached the optimal design and implementation of a solar PV–wind–biogas–VRFB storage integrated smart hybrid microgrid to ensure zero loss of power supply [8]. While the all-VRFB power storage system has many advantages, it is still limited by the narrow operating temperature range, low energy density, precipitation of vanadium dioxide, and expensive vanadium salt. The VRFB system presents poor thermal stability of V^5+^ electrolyte solution [9]. Y.K. Zeng et al. carried out a comprehensive comparison of the VRFB and ICRFB (iron/chromium) large-scale energy storage systems, and the ICRFB showed a lower cost than the VRFB [10]. N. Gurief et al. introduced a new concept of redistributing reactants within the flow frame to reduce the concentration overpotential and increase the limiting current density and cycle efficiencies [11]. These restrictions have prompted researchers to find a new reduced-cost RFB system. Thus, various RFBs have been developed, such as the V/Fe [12,13], V/Br [14], V/Ce [15], and V/I [16] semi-vanadium systems and Zn/Br [17], Zn/Fe [18], Zn/Ce [19], Ce/Pb [20], Fe/Pb [21], Fe/Cl [22], soluble all-lead (all-Pb) [23], and all-Fe RFB [24] non-vanadium systems. P.K. Leung and Z. Xie et al. [25,26,27,28,29], reviewed the developments and challenges of the Zn/Ce RFB, including single or mixed electrolytes, supporting electrolytes, additives, and the electrode and reaction mechanism. The Zn/Ce RFB has the potential to store a large amount of energy economically and efficiently because of the high thermodynamic open-circuit cell voltage. However, there are still numerous problems that must be overcome, such as the zinc deposition on the negative electrode, the evolution of oxygen during charging, and requirement of high chemical stability for a positive electrode. In addition, the supporting electrolyte plays an important role. By using sulfuric acid (H_2_SO_4_) as a supporting electrolyte with various concentrations and temperatures, different cerium ions can easily produce a variety of ion-combined coordination substances, which makes the electrochemical behavior of the Ce(III)/Ce(IV) redox pair more complicated [25,26,27]. G. Nikiforidis et al. used methanesulfonic acid (CH_3_SO_3_H) mixed with hydrochloric acid (HCl) as the supporting electrolyte for a positive Ce(III)/Ce(IV) redox pair that showed higher redox reversibility and a higher reduction current density than the pure CH_3_SO_3_H solution [27]. Z. Na [29] et al., using an acid-treated graphite carbon felt (PGF) electrode, achieved good electrocatalytic activity. In our previous research, we found that the C–TiO_2_–CoP and C–TiO_2_–PdO composite electrodes synthesized by the sol–gel process and electroless plating method can greatly improve the voltage efficiency of the RFB for non-vanadium electrolytes such as iodide and iron salts [13,16]. Compared with the all-vanadium RFB, the V/I RFB can reduce the cost of the vanadium salt, and the ascorbic acid can effectively increase the electrochemical reversibility of the iodide salt as shown by our previous study [16]. However, the V/I RFB system still has the problems of low standard potential (about 0.46 V) and expensive vanadium salts [16]. The main aim of this research was to develop a new Ce/ascorbic acid/I active species redox couple for the RFB design. Using the cerium nitrate hexahydrate Ce(NO_3_)_3_·6H_2_O salt with high reduction potential (1.28 to 1.72 V) from Ce(III) to Ce(IV), and ascorbic acid with low reduction potential (0.06 V) and high solubility, can assist the reversibility of the electrochemical reaction of the iodine solution [4,16,20]. A new non-vanadium Ce/ascorbic acid/I RFB system combined with the modification of the key materials such as electrodes and isolation membranes was designed. This system can obtain a higher standard potential and energy density than of the all-VRFB system and effectively reduce the costs.

## 2. Materials and Methods

### 2.1. Fabrication of the Composite Electrodes and the Modification of the Nafion 117 Membrane

We fabricated a series of composite electrodes including carbon paper–titanium dioxide (C–TiO_2_), carbon paper–palladium oxide (C–PdO), and carbon paper–titanium dioxide–palladium oxide (C–TiO_2_–PdO) from our previous study [13]. First of all, graphite carbon paper electrodes (C electrodes; Shenhe Carbon Fiber Materials Co. Ltd., Liaoning, China) were acid-treated and modified using the sol–gel and electroless plating methods to form various composite electrodes. The C–TiO_2_ electrode was fabricated using the tetrabutyric acid mixed with EtOH/HCl (pH = 1) aqueous solution with a tetrabutyric acid/EtOH/HCl_eq_ molar ratio of 1:8:4 in a flask, which was mechanically stirred to carry out the hydrolysis reaction at room temperature. The electroless plating solution was 19 g/L Na_2_C_4_H_4_O_4_·6H_2_O, 10 g/L PdCl_2_, 8.5 g/L HCl, and 25.6 g/L C_2_H_4_(NH_2_)_2_. The effective area of the electrode was 5 × 5 cm^2^. Finally, these composite electrodes were sintered at 400 °C in an oven for one hour. In addition, we modified a Nafion 117–SiO_2_–SO_3_H (N-117–SiO_2_–SO_3_H) membrane using 3-mercaptopropyl trimethoxysilane (MPTMS, Acros Organics, Bergen County, NJ, USA) and hydrogen peroxide (H_2_O_2_, SHIMAKYU, Japan) in our laboratory [30]. The Nafion 117(N-117) and Nafion 212 (N-212) membranes were purchased from DuPont Inc. (DuPont de Nemours, Inc., Wilmington, DE, USA). All chemicals used were of analytical reagent grade.

### 2.2. Electrocatalytic Activity Test of Composite Electrodes

The electrocatalytic activity of the composite electrode was evaluated using the hydrogen evolution reaction (HER). In addition, the exchange current density (I_o_), Tafel slope value (−b), overpotential (E_op_) were calculated [16]. The cyclic voltammetry (CV) experiments were conducted in a three-electrode cell with a CHI 6273 C electrochemical instrument (CH Instruments, Inc., Austin, TX, USA). The cathodic polarization curves were acquired at a constant negative potential and scan rate (1 mV/s) to measure a steady current value in 0.1 M CH_3_SO_3_H supporting electrolytes. The composition of the three-electrode cell was as follows: an Ag/AgCl electrode served as a reference electrode, a platinum gauze was used as a counter electrode, and a composite electrode with a surface area of 0.3 × 0.3 cm^2^ was used as a working electrode. The electrochemical properties, such as anodic current (I_a_), cathodic current (I_c_), anodic potential (E_a_), cathodic potential (E_c_), potential interval (ΔE_p_), and the ratio of the anodic/cathodic current (I_a_/I_c_), were obtained from the CV data [31,32].

### 2.3. Measurement of Electrochemical Characteristics of Active Species on Composite Electrodes

#### 2.3.1. Cerium Salt Active Species

The cerium nitrate hexahydrate Ce(NO_3_)_3_·6H_2_O, as an active species, was dissolved in solutions of 0.1 M CH_3_SO_3_H supporting electrolytes. The CV test was carried out on various composite electrodes in 0.01 M [Ce(NO_3_)_3_·6H_2_O]/0.1 M CH_3_SO_3_H solution. The electrochemical properties were defined from the CV test data.

#### 2.3.2. Iodine/Ascorbic Acid Active Species

The iodine (I_2_)/ascorbic acid solutions were obtained through dissolving the potassium iodate (KIO_3_) and potassium iodide (KI) in H_2_SO_4_ acidic reaction. The reaction equation is expressed as Equation (1).

(1) $${\mathrm{IO}}_{3}^{-}+5I^{-}+6H^{+}\to 3I_{2}+3H_{2}O$$

The electrochemical properties were assessed using the CV experiment for 1.0 M I_2_/2.0 M H_2_SO_4_ solutions with different ascorbic acid content.

### 2.4. Charge/Discharge Test

#### 2.4.1. Preparation of Positive and Negative Electrolytes

There were two active electrolytic solutions, 1.0 M Ce(NO_3_)_3_·6H_2_O) and 1.0 M I_2_, employed as positive and negative electrolytes. Both 2.0 M H_2_SO_4_ and 2.0 M CH_3_SO_3_H were used as the supporting electrolytes, respectively. The 1.0 M I_2_/ascorbic acid/2.0 M H_2_SO_4_ solutions were obtained through dissolving the KIO_3_ and KI in acidic reaction, as expressed in Equation (1), then adding 0.25 M ascorbic acid. All chemicals used were of analytical reagent grade.

#### 2.4.2. Cell Performance of a Single Ce/Ascorbic Acid/I RFB System by Modifying Electrode and Separation Membrane

The charge/discharge tests were carried out using 1.0 M Ce(NO_3_)_3_·6H_2_O/2.0 M CH_3_SO_3_H as the positive electrolyte, 1.0 M I_2_/ascorbic acid/2.0 M H_2_SO_4_ as the negative electrolyte, C–TiO_2_–PdO as both positive and negative electrodes, and Nafion 117–SiO_2_–SO_3_H as the separation membrane at a current density of 20 mA/cm^2^ and using 20 mL of electrolyte solutions. A charge/discharge test was conducted using a WBCS3000 battery cycler system (Top Trans, Korea) and CT2001C 10 V/2A (Wuhan Land Co., Wuhan, China) apparatus.

## 3. Results and Discussion

### 3.1. Electrocatalytic Characteristics of Composite Electrodes

In this experiment, the electroless plating and sol–gel methods were used to fabricate the composite electrodes, which were then sintered at 400 °C. The crystal structure identification and surface morphology analysis of the composite electrodes were conducted according to the XRD diffraction patterns and the SEM/EDS in our previous studies [16]. The electrolysis of aqueous electrolyte produces hydrogen gas (H_2_), which is defined as the HER. The HER requires an electrocatalyst where reaction kinetics and electrode stability are prime factors.

The typical Tafel polarization curve was proposed in 1905, and an empirical formula relating overpotential (E_op_) and electrochemical reaction current (I) is expressed in Equation (2).

E = a + b log I(2)

The diagram of polarization potential (E) vs. log I is called the Tafel curve; the b value is the slope of the straight line, which is called the Tafel Slope. From the slope, the electron transfer coefficient (a) can be obtained, and the intercept (E = 0) can obtain the exchange current (I_o_). Overpotential (E_op_) is the difference between the electrode potential when an electrode reaction deviates from the equilibrium and the equilibrium potential of this electrode reaction, i.e., the potential difference between no current passing (under equilibrium) and current passing [33,34]. The occurrence of hydrogen gas on the electrode was an active polarization phenomenon. The HER is usually used to evaluate the electrocatalytic activity of metal/alloy or non-metallic element composite materials (such as NiP, Co–Mo–TiO_2_, and conductive polymers) [33]. The linear polarization curve has been widely identified as one of the most important analysis tools for determining the HER kinetic parameters [34]. The exchange current density (I_o_), Tafel slope value (−b), overpotential (E_op_), and transmission coefficient (a) can be calculated to determine the electrocatalytic effect, active surface area, and electrode stability [13]. Simply stated, for a good metal electrocatalytic mechanism, at a low E_op_, the rate determination step (rds) is determined by the Tafel equation. At a higher E_op_, the rds is dominated by the Volmer equation and the Heyrovsky equation for secondary discharge, as shown in Equations (3)–(5). The rds is determined by the Tafel equation if the value (−b) is near 30 mV/dec; in general, the metal electrode belongs to this model. However, when the value is near 120 mV/dec, the rds is decided by the Volmer and Heyrovsky equations, and the composite electrode might be better suited to the other two models [13,35].

(3) $$\mathrm{Volmer}\;\mathrm{equation}:{\;H}_{3}O^{+}+{\;e}^{-}+*\;\to {\;H}^{*}{+H}_{2}O$$

(4) $$\;\;\;\mathrm{Heyrovsky}\;\mathrm{equation}:\;\;\;{\;H}^{*}+H_{3}O^{+}+{\;e}^{-}\;\to {\;H}_{2}+{\;H}_{2}O$$

(5) $$\mathrm{Tafel}\;\mathrm{equation}:2H^{*}\to {\;H}_{2}$$

where * represents the active site on the electrode surface, and H* represents a hydrogen atom adsorbed on the active site. Generally, the one step that kinetically limits the electrochemical reaction is called the rds [35].

**Figure 1:**
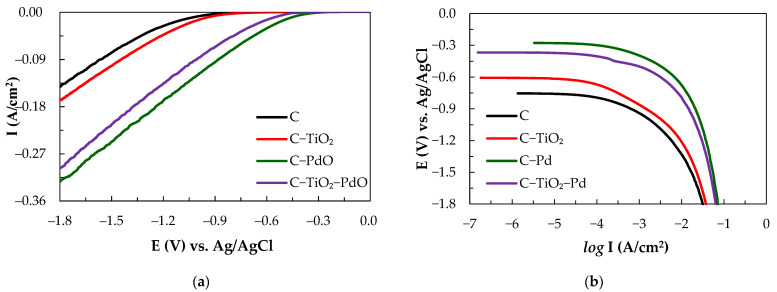
CV tests for (**a**) cathodic polarization curves and (**b**) linear polarization curves recorded on various electrodes in 0.1 M CH_3_SO_3_H solution at a scan rate of 1 mV/s.

Figure 1a shows the steady-state cathodic polarization curves of the various composite electrodes in methanesulfonic acid (0.1 M CH_3_SO_3_H) at a scan rate of 1 mV/s and the corresponding linear polarization curves. The kinetic parameters are as shown in Figure 1b and Table 1. The results show that the C–PdO electrode has a low E_op_ and the highest exchange current density, with an I_o_ value of 4.2 μA/cm^2^, and presents the best electrocatalytic properties. The order of the electrocatalytic activity was C–PdO > C–TiO_2_–PdO > C–TiO_2_ > C from the exchange current density value. All electrodes exhibit a single-stage Tafel slope (−b) value in the CH_3_SO_3_H solution. At the higher E_op_ values between −300 and −900 mV, the (−b) values of the C, C–TiO_2_, C–PdO and C–TiO_2_–PdO electrodes are 120, 169, 79, and 88 mV/dec, respectively. The C–TiO_2_–PdO electrode has the lowest value. The rds was dominated by the Volmer and Heyrovsky equations [13,35].

### 3.2. Electrochemical Characteristics of Active Electrolytes on Composite Electrodes

#### 3.2.1. Cerium Salt Active Electrolyte

**Figure 2:**
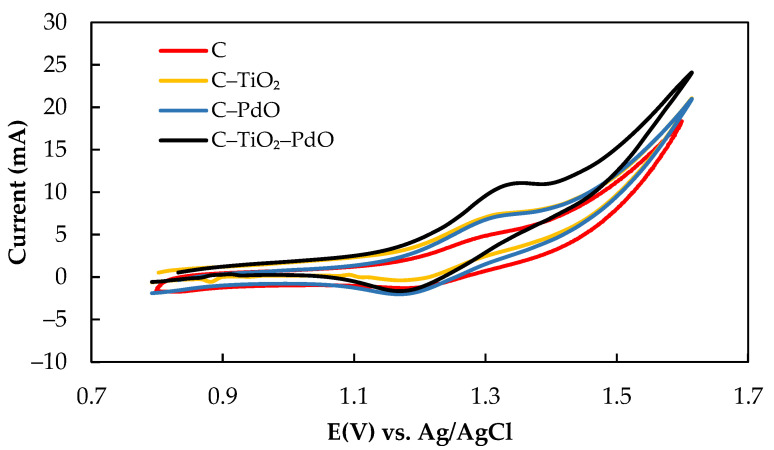
Cyclic voltammograms for 0.01 M Ce(NO_3_)_3_·6H_2_O/0.1 M CH_3_SO_3_H solution.

The electrochemical characteristics of the Ce (III)/Ce (IV) active species and CH_3_ SO_3_H supporting electrolyte on various composite electrodes were investigated using the CV test. The cyclic voltammograms of various electrodes in 10 mL of 0.01 M Ce(NO_3_)_3_·6H_2_O/0.1 M CH_3_SO_3_H solution at a scanning rate of 20 mV/s in the range of 0.7 to 1.7 V are as presented in Figure 2, and the electrochemical characteristics are as summarized in Table 2. The values of I_a_ and I_c_ were high, indicating excellent redox efficiency of the composite electrodes in the Ce(NO_3_)_3_·6H_2_O/CH_3_SO_3_H solutions. The I_a_ values were in the following order: C–TiO_2_–PdO (4.24 mA) > C–PdO (4.22 mA) > C (3.59 mA) > C–TiO_2_ (3.40 mA) for Ce(NO_3_)_3_·6H_2_O/CH_3_SO_3_H solutions. The potential interval values (ΔE_p_) were in the following order: C–TiO_2_ (0.18 V) > C–TiO_2_–PdO (0.15 V) > C (0.14 V) > C–PdO (0.13 V). The high ΔE_p_ value demonstrated that the energy barriers of the redox reactions were higher because of the effects of the Ce(NO_3_)_3_·6H_2_O/CH_3_SO_3_H electrolyte solutions on the tested electrodes. The C–PdO electrode presented a better electrocatalytic activity than the C–TiO_2_–PdO electrode according to the HER kinetic parameter analysis, but the ratio of the anodic–cathodic current (I_a_/I_c_) was 3.13. By comparison, the C–TiO_2_–PdO electrode exhibited a higher anodic/cathodic current and a more symmetric I_a_/I_c_ value (1.09), near unity, which indicated a quasi-reversible redox reaction in the Ce(NO_3_)_3_·6H_2_O/CH_3_SO_3_H solutions. In addition, the TiO_2_ particles between a carbon matrix and the Pd metal/Pd oxide layer increased the active surface area of the electrode, improving the electrocatalytic effect of the electrode [13]. Consequently, the C–TiO_2_–PdO electrode performed most favorably among all the electrode types in the redox kinetic reactions.

#### 3.2.2. Iodine/Ascorbic Acid Active Species

**Figure 3:**
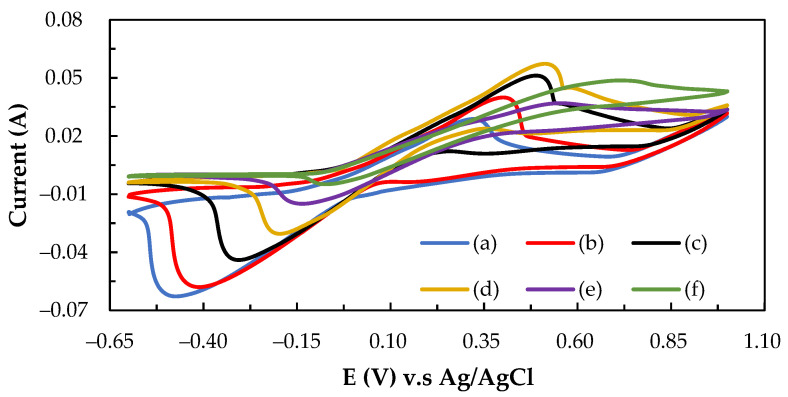
The effect of adding ascorbic acid on the electrochemical properties of I_2_/ascorbic acid/H_2_SO_4_ active electrolyte (**a**) 1.0/0.0/2.0 M (without ascorbic acid) (**b**) 1.0/0.18/2.0 M (**c**) 1.0/0.36/2.0 M (**d**) 1.0/0.54/2.0 M (**e**) 1.0/0.72/2.0 M (**f**) 0.0/1.0/2.0 M (without I_2_ solution).

**Figure 4:**
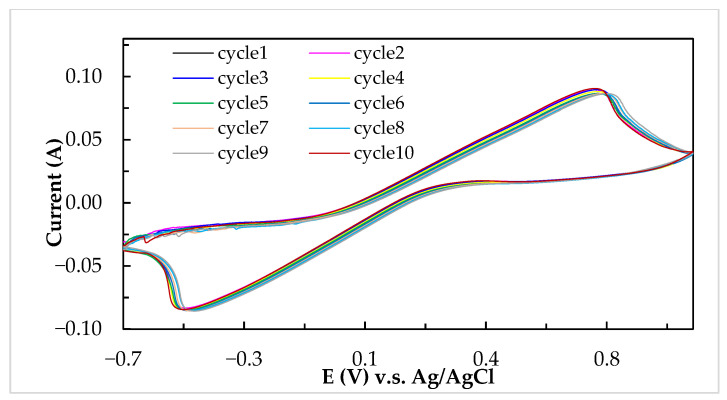
Cyclic voltammograms of I_2_/ascorbic acid/H_2_SO_4_ active electrolyte at a concentration ratio of 1.0/0.18/2.0 M for 10 cycles at a scanning rate of 10 mV/s.

Ascorbic acid (C_6_H_8_O_6_) with low reduction potential (+0.06 V) easily occurs in the oxidation reaction to form the dehydroascorbic acid (C_6_H_6_O_6_). On the contrary, the iodine (I_2_) electrolyte with high reduction potential (+0.54 V) was more suitable to the reduction reaction to form the anion (I^−^). In previous literature [36], the experiments demonstrated that vitamin C can be utilized in a reversible redox reaction consisting of its reduced (ascorbic acid), radical (semidehydroascorbic acid), and oxidized (dehydroascorbic acid) forms using enzymatic (by ascorbate oxidase) and non-enzymatic (by iodine) reactions. Previous research confirmed that the C–TiO_2_–PdO composite electrode showed good electrochemical activity in the V/I RFB [16]. Figure 3 shows the CV curves with the addition of ascorbic acid into the iodine electrolyte solutions for the C–TiO_2_–PdO composite electrode and the related electrochemical characteristics, as summarized in Table 3. The oxidation current (I_a_, 27 mA) of iodine electrolyte solution without adding ascorbic acid was less than the reduction current (I_c_, 40 mA), so the ratio of I_a_/I_c_ value (0.68) was much smaller than the unity value. The oxidation current of iodine solution with added ascorbic acid increased, and the I_a_/I_c_ value was close to unity, at 0.18 (0.96) and 0.36 M (1.04), respectively. However, the reduction current of the iodine solution decreased when the concentration of ascorbic acid was more than 0.36 M, and the I_a_/I_c_ deviated more than the unity value. This means that this redox reaction was an irreversible trend, and the optimum ascorbic acid amount was between 0.18 and 0.36 M, as listed in Table 3. In addition, from Figure 4, it can be seen that the I_2_/ascorbic acid/H_2_SO_4_ active electrolyte at molarity ratio of 1.0/0.18/2.0 M for 10 cycles shows a good cyclic stability of the reversible redox reaction.

### 3.3. Cell Performances

**Figure 5:**
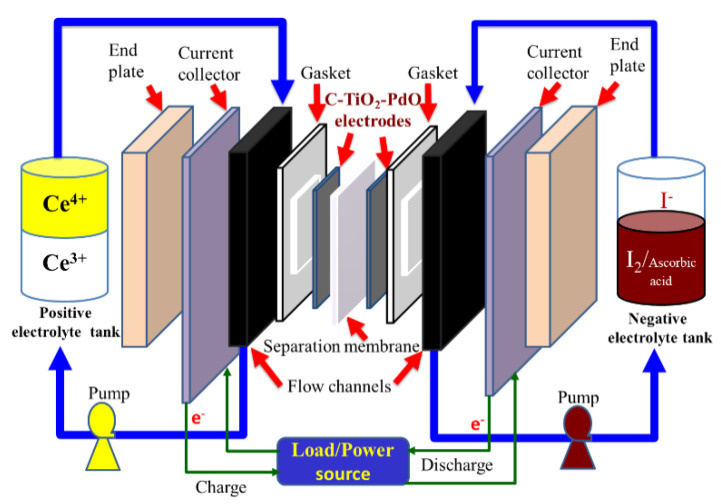
Schematic of the Ce/ascorbic acid/I RFB under a charge–discharge cycle.

**Figure 6:**
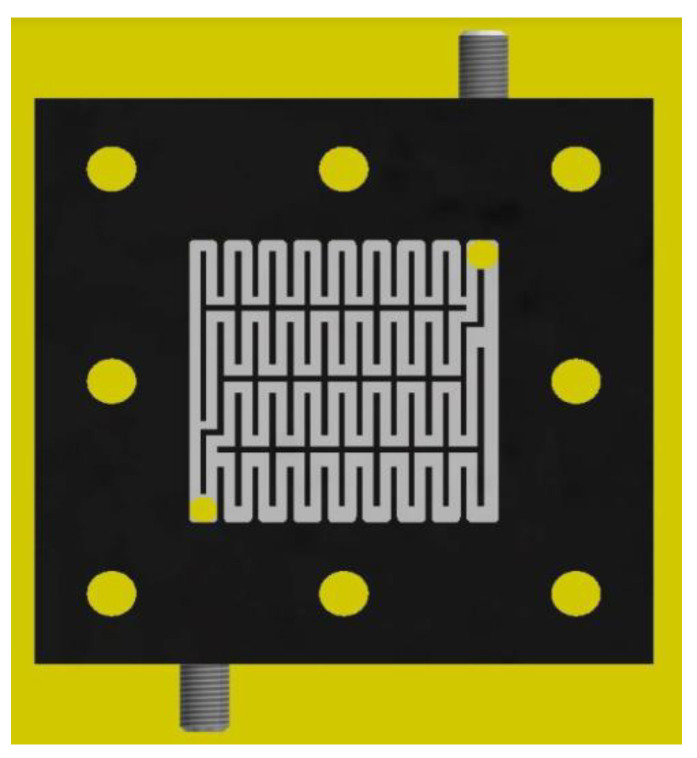
Schematic depiction of staggered-type flow channels in the Ce/ascorbic acid/I RFB system.

We designed a single cell for the Ce/ascorbic acid/I RFB system. This cell comprised a pair of electrodes, a separation membrane, a pair of flow channels, and a pair of current collectors similar to our previous study of the RFB system [13], as shown in Figure 5. A schematic depiction of flow channels is given in Figure 6.

The overall reactions of the positive and negative redox couples electrolytes for the Ce/ascorbic acid/I RFB system can be expressed as Equations (6)–(10).

Positive electrode:

(6) $$2{\mathrm{Ce}}^{4+}{+2e}^{-}\overset{\mathrm{discharge}}{\underset{\mathrm{charge}}{⇄}}2{\mathrm{Ce}}^{3+}\;\;\;\;{\;E}^{o}=+1.72\;V\;\mathrm{vs}.\;\mathrm{SHE}$$

Negative electrode:

(7) $$2I^{-}\overset{\mathrm{discharge}}{\underset{\mathrm{charge}}{⇄}}{\;I}_{2}{+2e}^{-}\;\;\;\;\;\;\;\;{\;E}^{o}=0.54\;V\;\mathrm{vs}.\;\mathrm{SHE}\;$$

(8) $$C_{6}H_{8}O_{6}\overset{\mathrm{discharge}}{\underset{\mathrm{charge}}{⇄}}C_{6}H_{6}O_{6}\;+\;2H^{+}\;+\;{2e}^{-}\;\;\;\;{\;E}^{o}=0.06\;V\;\mathrm{vs}.\;\mathrm{SHE}\;$$

Overall reactions:

(9) $$2{\mathrm{Ce}}^{4+}\;+\;2I^{-}\overset{\mathrm{discharge}}{\underset{\mathrm{charge}}{⇄}}2{\mathrm{Ce}}^{3+}{\;+\;I}_{2}\;\;\;\;\;\;\;\;{\;E}_{\mathrm{cell}}^{o}=1.18\;V$$

(10) $$2{\mathrm{Ce}}^{4+}{\;+\;C}_{6}H_{8}O_{6}\overset{\mathrm{discharge}}{\underset{\mathrm{charge}}{⇄}}\;\;2{\mathrm{Ce}}^{3+}{\;+\;C}_{6}H_{6}O_{6}{+2H}^{+}\;\;\;\;{\;E}_{\mathrm{cell}}^{o}=1.66\;V$$

Therefore, the standard electromotive forces ${\;E}_{\mathrm{cell}}^{o}$ of the Ce/ascorbic acid/I RFB system were between 1.18 and 1.66 V in the discharging mode.

#### 3.3.1. Cell Performance of a Single Ce/Ascorbic Acid/I RFB System with Different Supporting Electrolyte

**Figure 7:**
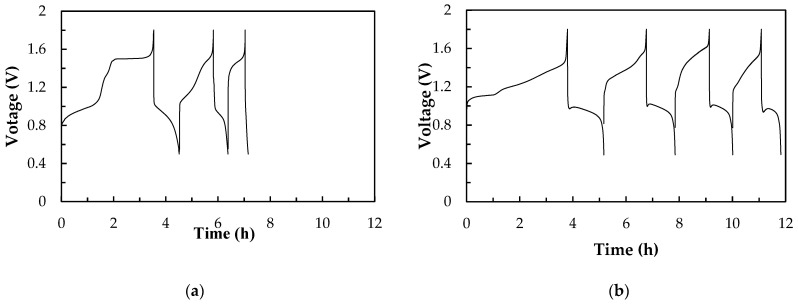
Charge–discharge diagrams of a Ce/ascorbic acid/I RFB system with H_2_SO_4_ or CH_3_SO_3_H as positive supporting electrolyte (**a**) 1.0 M Ce(NO_3_)_3_/2.0 M H_2_SO_4_ (**b**) 1.0 M Ce(NO_3_)_3_/2.0 M CH_3_SO_3_H. Other components: 1.0 M I_2_/0.20 M ascorbic acid/2.0 M H_2_SO_4_ as a negative electrolyte, Nafion 117 as a membrane, and carbon papers as positive/negative electrodes, as well as two staggered-type flow channels at a current density of 20 mA/cm^2^ and 20 mL of electrolyte solutions.

Figure 7 shows the charge/discharge diagram of a Ce/ascorbic acid/I RFB system with the staggered-type flow channels at the current density of 20 mA/cm^2^. The unmodified Nafion 117 and carbon paper were used as a separation membrane and positive/negative electrodes, respectively. The 1.0 M I_2_/2.0 M H_2_SO_4_ solutions were used as a negative active electrolyte with 2.0 M H_2_SO_4_ as a supporting electrolyte. The positive active electrolyte was 1.0 M Ce(NO_3_)_3_ with 2.0 M H_2_SO_4_ or 2.0 M CH_3_SO_3_H as a supporting electrolyte, as presented in Figure 7a,b, and the performance data are summarized in Table 4. The longer charging curve in the first round may be related to the HER effect of the positive electrode during charging. It can be clearly seen from Figure 7a that the efficiency and capacity of the battery have obviously declined over time, and the battery has serious leakage when it runs to the third lap, so it cannot be charged and discharged smoothly. We found that the white precipitates were deposited on the electrodes and the flow channels; it was known from the literature that this precipitate was cerium oxide [37]. This system cannot be charged or discharged because the reduction of the active electrolyte causes a rapid decrease in the battery capacity. Figure 7b is the charge–discharge diagram of a Ce/ascorbic acid/I RFB system with 2.0 M CH_3_SO_3_H as a supporting electrolyte for the 1.0 M Ce (NO_3_)_3_ active electrolyte. The HER effect can also be seen; however, the capacity degradation of the battery was less serious than that of the H_2_SO_4_ supporting electrolyte. Compared with the sulfuric acid, it can be clearly found that the CE, VE, and EE values after the third lap were about 68%, 65%, and 46%, and the discharge capacity was improved from 36 to 263 mAh. Therefore, the methanesulfonic acid (CH_3_SO_3_H) was more suitable as the supporting electrolyte of the Ce (NO_3_)_3_ active electrolyte, but the key materials still need to be modified to obtain a suitable Ce/ascorbic acid/I RFB system.

#### 3.3.2. Cell Performances of a Single Ce/Ascorbic Acid/I RFB System by Modifying Electrode and Separation Membrane

**Figure 8:**
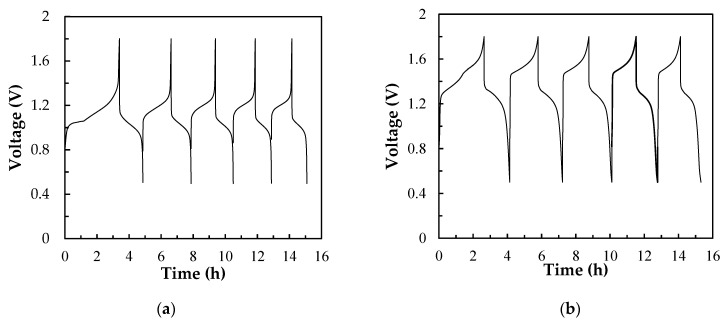
The charge/discharge diagrams of a Ce/ascorbic acid/I RFB system with 1.0 M Ce(NO_3_)_3/_2.0 M CH_3_SO_3_H as a positive electrolyte, 1.0 M I_2_/0.25 M ascorbic acid/2.0 M H_2_SO_4_ as a negative electrolyte and C–TiO_2_–PdO composite electrodes as positive/negative electrodes, with two serpentine-type flow channels at a current density of 20 mA/cm^2^ and 20 mL of electrolyte solutions with (**a**) N-117 (**b**) modified N-117–SiO_2_–SO_3_H as a separation membrane.

**Figure 9:**
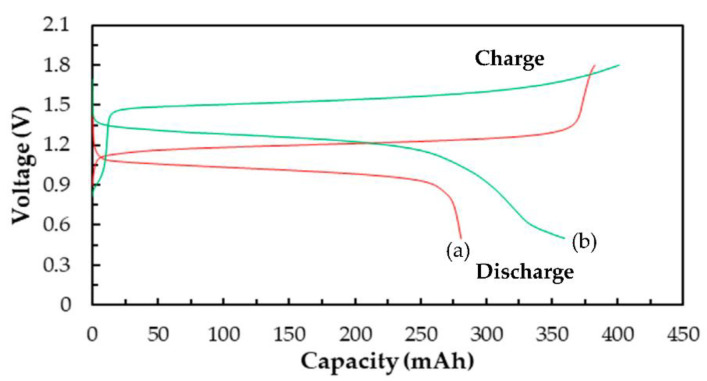
The charge and discharge capacity curves of the third cycle of the Ce/ascorbic acid/I RFB system with different separation membranes: (**a**) N-117, (**b**) N-117–SiO_2_–SO_3_H.

Figure 8 shows the charge/discharge cycle diagrams of the Ce/ascorbic acid/I RFB system in the third lap with N-117 and the modified N-117–SiO_2_–SO_3_H as a separation membrane. The C–TiO_2_–PdO composite electrodes were used as the positive and negative electrodes. The electrochemical activity of the C–TiO_2_–PdO composite electrodes was better than that of the carbon paper (C electrode) and showed a good cell performance; the VE% increased from 65% to 79%, and the EE% was raised from 44% to 58%, as shown in Figure 8a and Table 4. In order to improve the ion cross-contamination, using the modified N-117–SiO_2_–SO_3_H membranes to replace the commercial N-117 can effectively enhance the cell efficiency, as shown in Figure 8b. Particularly, the CE% of the modified N-117–SiO_2_–SO_3_H membrane was raised from 73% to 90%, and the EE% increased from 44% to 72%, as listed in Table 4. In addition, from the charge and discharge capacity curve of Figure 9, it can clearly be seen that the standard potential (${\;E}_{\mathrm{cell}}^{o}$) and capacity exhibited an improved tendency after replacing the N-117 separation membrane, and the data are summarized in Table 4. The ${\;E}_{\mathrm{cell}}^{o}$ and the capacity of the single Ce/ascorbic acid/I cell were 1.20 V/280 mAh (N-117) and 1.46 V/360 mAh (N-117–SiO_2_–SO_3_H), respectively. The results mean that the modified N-117–SiO_2_–SO_3_H membrane can effectively inhibit the ion cross-contamination, thus, the standard potential of this Ce/ascorbic acid/I RFB system significantly increased from 1.20 to 1.46 V and the discharge capacity from 280 to 360 mAh. A plausible explanation for these results is that the modified membrane with low permeability/water uptake and high ion exchange capacity (IEC) value not only inhibits the cross-over pollution of the active species ions and avoids the non-equivalent concentration effect of the Nernst equation but also increases the conductivity of H^+^ protons [30]. Thus, the modified membrane can help to effectively enhance the standard potential and discharge capacity of this Ce/ascorbic acid/I RFB system.

**Figure 10:**
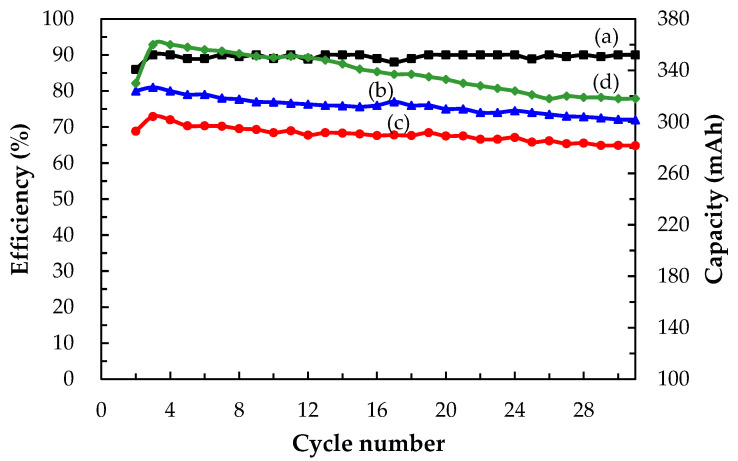
Cyclic efficiency and capacity in terms of (**a**) CE, (**b**) VE, (**c**) EE, and (**d**) discharge capacity of a Ce/ascorbic acid/I RFB system with modified N-117–SiO_2_–SO_3_H as the separation membrane and staggered-type flow channels, 1.0 M Ce(NO_3_)_3_/2.0 M CH_3_SO_3_H as a positive electrolyte, 1.0 M I_2_/0.25 M ascorbic acid/2.0 M H_2_SO_4_ as a negative electrolyte, and C–TiO_2_–PdO composite electrodes as positive/negative electrodes at a current density of 20 mA/cm^2^ and 20 mL of electrolyte solutions.

Figure 10 shows charge–discharge performance over 30 cycles (2nd to 31st) in terms of (a) CE, (b) VE, (c) EE, and (d) discharge capacity of a single Ce/ascorbic acid/I RFB system. The results showed that the VE and discharge capacity values were decreased with the increase of the charge/discharge cycle number because of factors such as internal ohmic resistance, overcharge, and concentration polarization. The average CE, VE, and EE of this Ce/ascorbic acid/I RFB system were 89.51%, 75.88%, and 68.04% after 30 cycles, and the average discharge capacity, was 337.67 mAh. The attenuated percentage was less than 6% for the EE value and discharge capacity.

For large-scale commercial applications, the stacks of the RFB system are often fabricated in series and parallel cross combinations to enhance storage capacity and control the voltage of the battery module. The Ce/ascorbic acid/I RFB system with high standard potential and low-cost electrolyte shows a potential for a KWh-scale energy storage system application via the assembly of the battery stacks.

## 4. Conclusions

The TiO_2_ layer of the C–TiO_2_–PdO composite electrode disperses the Pd metal particles, which reduces the metal particles agglomeration and increases the active area of the C–TiO_2_–PdO composite electrode. The PdO formed after sintering for the C–TiO_2_–PdO composite electrode has excellent electrocatalytic properties. The steady-state cathodic polarization curve data showed a lower overpotential (E_op_) and a higher exchange current density (I_o_) in the methanesulfonic acid than that of the carbon paper electrode (C). In addition, the novel Ce/ascorbic active/I active species have excellent electrochemical activity on this composite electrode. The most suitable reversibility of oxidation/reduction reactions was obtained when the molarity ratio of the iodine solution/ascorbic acid was about 1/0.25. In the Ce/ascorbic acid/I RFB system, the modified N117–SiO_2_–SO_3_H as a separation membrane inhibits the ion cross-contamination, and the C–TiO_2_–PdO composite electrodes enhance the electrocatalytic activity. Therefore, this Ce/ascorbic acid/I RFB system presented good cell performance due to the modified key materials, which greatly increased the EE% value by 64%, the standard potential by 27%, and the discharge capacity by 37%. The attenuated percentage was less than 6% for the EE value and discharge capacity.

## Figures and Tables

**Table 1:** The HER kinetic parameter analysis of the linear polarization curves for various electrodes in 0.1 M CH_3_SO_3_H solution.

Electrode	−b	I_o_	E_eq_/E_op_	I
	(mV/dec)	(μA/cm^2^)	−(mV) at −10 (mA/cm^2^)	(mA/cm^2^) at −900 (mV)
C	120	0.25	754/1034	2.87
C–TiO_2_	169	0.85	606/947	6.5
C–PdO	79	4.2	276/458	95.38
C–TiO_2_–PdO	88	2.69	367/560	66.09

**Table 2:** Electrochemical characteristics of 0.01 M Ce(NO_3_)_3_·6H_2_O solution on various electrodes with 0.1 M CH_3_SO_3_H as a supporting electrolyte.

Electrode	E_a_ (V)	E_c_ (V)	ΔE_p_ (V)	I_a_ (mA)	I_c_ (mA)	I_a_/I_c_
C	1.31	1.17	0.14	3.59	2.47	1.45
C–TiO_2_	1.30	0.12	0.18	3.40	2.05	1.31
C–PdO	1.29	1.16	0.13	4.22	1.35	3.13
C–TiO_2_–PdO	1.33	1.18	0.15	4.24	3.89	1.09

**Table 3:** Electrochemical characteristics of I_2_/ascorbic acid/H_2_SO_4_ solution on C–TiO_2_–PdO composite electrode with 2.0 M H_2_SO_4_ as a supporting electrolyte.

Electrode	Electrolyte I_2_/Ascorbic Acid/H_2_SO_4_Conc. (M)	E_a_ (V)	E_c_ (V)	ΔE_p_ (V)	I_a_ (mA)	I_c_ (mA)	I_a_/I_c_
C–TiO_2_–PdO	(a) 1.0/0.00/2.0 M	0.31	−0.43	0.74	27	40	0.68
	(b) 1.0/0.18/2.0 M	0.48	−0.19	0.67	44	46	0.96
	(c) 1.0/0.36/2.0 M	0.51	−0.24	0.75	50	48	1.04
	(d) 1.0/0.54/2.0 M	0.54	−0.20	0.74	38	28	1.36
	(e) 1.0/0.72/2.0 M	0.70	−0.47	1.17	44	20	2.2
	(f) 0.0/1.00/2.0 M	0.75	−0.05	0.80	44	19	2.31

**Table 4:** The performance of the Ce/ascorbic acid/I RFB system with various key components at the third lap charge–discharge test.

Key Materials	Types	CE (%)	VE (%)	EE (%)	${\;E}_{\mathrm{cell}}^{o}$ (V)	Discharge Capacity (mAh)
Supporting electrolyte *	H_2_SO_4_	18	51	9	1.25	36
	CH_3_SO_3_H	68	65	44	1.15	263
Electrode *	C–TiO_2_–PdO	73	79	58	1.20	280
Separation membrane	N-117–SiO_2_–SO_3_H	90	81	72	1.46	360

* Separation membrane: Nafion 117.

## Data Availability

The data presented in this study are available on request from the corresponding author.

## Sample Availability

The data presented in this study are available on request from the corresponding author.
